# Dialogic Reading to Empower Executive Functions in Preschoolers

**DOI:** 10.3390/children8050373

**Published:** 2021-05-09

**Authors:** Costanza Ruffini, Silvia Spoglianti, Clara Bombonato, Silvia Bonetti, Maria Chiara Di Lieto, Chiara Pecini

**Affiliations:** 1 Department of Education, Languages, Intercultures, Literatures and Psychology (FORLIPSI), University of Florence, Via di San Salvi 12, 50135 Firenze, Italy; costanza.ruffini@unifi.it (C.R.); chiara.pecini@unifi.it (C.P.); 2 Paroleincerchio, Centro per l’Età Evolutiva, Via Leopardi 90, 40026 Imola, Italy; silviaspoglianti@yahoo.it; 3 Department of Neuroscience, Psychology, Drug Research and Child Health, Viale Pieraccini 6, 50139 Firenze, Italy; cbombonato@unifi.it; 4 Equipe Multiprofessionale Evolutiva, Via della Vetraia 7, 55049 Viareggio, Italy; silviabonetti@hotmail.it; 5 IRCCS Stella Maris Foundation, Viale del Tirreno 341, 56128 Calambrone, Italy

**Keywords:** executive functions, dialogic reading, preschool, intervention, shifting

## Abstract

Among the interventions recently developed to enhance Executive Functions (EFs) in preschoolers, Quincey Quokka’s Quest (QQQ) is an illustrated book proposing EF activities embedded within a shared reading framework (Howard et al., 2017). In the present study, the Italian version of QQQ (QQQ_IT_) was tested in 20 typical developing 4–5 year old children. Standardized tests were used to assess EFs pre- and post- intervention. QQQ_IT_ was conducted once a week for 8 weeks in small groups. A positive trend was registered in QQQ_IT_ performances from the first to the last sessions and a significant improvement, in comparison to the control condition, was obtained in the Color and Form Game test. These results, supporting the feasibility of the QQQ_IT_ intervention and its efficacy in increasing shifting abilities, confirm the usefulness of ecological interventions to empower specific EF components in preschool contexts.

## 1. Introduction

Executive Functions (EFs) are a family of high-level cognitive functions activated by new and complex tasks when it is not sufficient to rely on instinct or automated activities. EFs allow the management of all other cognitive functions and consciously control thoughts and emotions [1]; they represent an evolutionary advantage for human beings [2]. The fractional model proposed by Miyake [3] and recently revised by Friedman and Miyake [4] identifies three main separated and interrelated functions: inhibition, updating and shifting. *Inhibition* consists of resisting interferences, impulses or strong desires to do something. Thanks to this process, we can choose how to react regardless of internal or external stimuli, to keep attention focused on tasks and to avoid distracting stimuli. *Updating* works on stimuli kept in memory for a limited time allowing mental operation on them. *Shifting* is the ability to change perspective, disengage attention to a habitual stimulus and avoid fixing on responses or thoughts.

The greatest changes in executive functioning occur in the preschool period when the main basic EF components develop. Inhibition, updating and shifting emerge from infancy up to first school years and are the grounds for higher-order EFs, such as problem solving and planning [5].

EFs, in particular the three main basic components, represent transversal processes that directly and indirectly predict school readiness, learning skills and academic success [6]. In the school context, inhibition supports appropriate behaviors within formal classes, allows to focus attention on relevant stimuli, including alphanumeric ones, and enables to promote and maintain goal-directed behaviors to complete tasks and homework. Working memory allows maintenance of the memory rules and cognitive instructions needed to learn and behave properly within the school context, it helps to link and update different information during text comprehension and problem solving and it represents an obligatory step for long-term memory consolidation and skill automatization. Shifting is the basis of logical and inferential thinking, it allows children to explore different perspectives and strategies and it is needed to adapt to different school subjects, tasks and instructions [5,7].

In order to empower these important cognitive processes, several studies developed EF interventions for early childhood through activities that, as suggested by Diamond and Lee [8,9], try to satisfy novelty, enjoyability and challenges. Two main types of training can be distinguished. The first type of intervention promotes EFs by involving children in intensive and structured activities specifically addressed to the main EF components such as inhibition, updating and shifting, simultaneously or separately trained. These interventions often provide computer-based training and algorithms that automatically change the level of task difficulty according to the child’s performance. However, in such interventions the child is required to work at a computer alone in a quiet place, involving high economical costs and not easily fitting with the school routine activities [10,11,12]. Due to these features, the effects of such training is hardly generalizable both to tasks that are not directly involved in the training and to daily life behaviors [8]. For example, CogMed Working Memory Training (RoboMemo^®^, CogMed Cognitive Medical Systems AB, Stockholm, Sweden), an evidence-based tele-rehabilitation software, proved effective in improving performance in working memory tasks similar to those proposed in the software but may show scarce generalization effects on other related neuropsychological and academic skills [13].

The second type of intervention is based on a multi-componential model of self-regulation where, besides activities on emotional and behavioral self-regulation, EF exercises are proposed to empower cognitive self-regulation [14]. These interventions act also on the environment by promoting responsive relationships, in which the adult (teacher or parent) mediates the acquisition of regulatory skills, structures adequate learning environments and provides models of self-regulation competencies [15]. They involve specific training for teachers and parents in order to promote knowledge on cognitive constructs and create adequate learning environments where gains in executive functions and self-regulation may be optimized and generalized [14,16,17,18,19]. Among the paper-and-pencil tasks based on the multi-componential model of self-regulation in preschoolers, different intervention programs have been developed. Traverso and collaborators, for example, implemented and experimented a training (Chicco and Nanà) that was effective in improving the ability to delay gratification, control impulsive responses, update information and suppress interference, with long-term effects on learning skills [20,21]. Another known intervention is the Promoting Alternative Thinking Strategies (PATHS) curriculum that produced significant effects on self-regulation, social and emotional skills and long-term benefits on academic achievement and cognitive performances [17,22]. Furthermore, Howard and collaborators involved several Australian centers for preschoolers in the Preschool Situational Self-Regulation Toolkit (PRSIST) program. PRSIST became highly acceptable by educators and children within their school context and daily routine and proved effective in enhancing EFs, self-regulation and school readiness, even if the latter two components did not reach significance compared to the control groups [14].

As mentioned before, a central aspect that differentiates computer-based training and interventions based on multi-componential models of self-regulation at school is the scarcity of transfer effects recorded in the first case in favor of more generalizable effects, with gains in functionality in terms of socio-emotional regulation in the second case [23].

Within the second type of intervention, recent studies promoted EF empowering embedded in daily life activities that, in comparison to computer-based training, are more ecological and suitable for younger children. Quincey Quokka’s Quest (QQQ) [24,25] is an illustrated book created with the aim of enhancing EFs within one of the most spontaneous and enjoyable activities of preschoolers: dialogic reading. QQQ tells in rhyme the adventures of a famous Australian quokka, traditionally defined as the “happiest animal in the world”. In each adventure, one or more EF components are challenged as the activities, proposed in the form of flexible games and with increasing difficulty, are specifically designed to stimulate inhibition, updating and shifting combining visual, spatial and verbal information processing modalities. It is low cost and suitable for different contexts, such as school or family settings. During QQQ, adults have a central role in strengthening children’s EFs; nevertheless, group working, learning by observation, peer monitoring are also involved. Howard and colleagues [25] used the QQQ book with children aged between 3 and 6 years and measured outcomes on inhibition (Go/No Go test), updating in visuospatial working memory (Mr Ant test) and shifting (Card sorting test). The training was conducted in small groups of children in three different conditions: twice a week for 5 weeks, once a week for 7 weeks and once a week for 9 weeks. The results supported that, regardless of the different conditions, QQQ was a highly promising EF intervention, with significant and long-term effects on visuospatial working memory and shifting.

To the best of our knowledge, QQQ is the first and unique dialogic reading book specifically addressed to empower EFs according to a fractionated model. Indeed, the proposed activities cannot be made up on commercial or classical books as they need to be pre-planned on the basis of the main targeted EF component. Although QQQ has been written in English and used with Australian children, thanks to its graphic, stylistic, and methodological characteristics, it could be implemented in different countries. The present study aimed to verify the feasibility and to measure, at a pilot level, the efficacy of the Italian version of Quincey Quokka’s Quest (QQQ_IT_) in a preschool sample.

## 2. Materials and Methods

### 2.1. Participants

Twenty Italian children (8 females and 12 males, aged 4–5 years, M: 57.6 months, SD: 6.9), attending a kindergarten school in Cecina (Livorno) were enrolled. All parents signed written informed consent. One child didn’t complete the intervention, and another was absent at the post training assessment. Therefore, analyses on QQQ_IT_ feasibility and efficacy were conducted on eighteen children (7 females and 11 males; M: 57.83 months, SD: 7.01).

### 2.2. Measures

The measures used in the present study include questionnaires completed by parents and direct EFs tests administered to the children.

#### 2.2.1. Questionnaires

Parents’ socio-economic and cultural status (SES), demographic data and the child’s main developmental milestones were collected by a questionnaire. No child had a history of pre-perinatal, sensorimotor or neurodevelopmental disorders.

In order to detect children with alterations in executive functioning, parents completed also the Preschool version of the Behavior Rating Inventory of Executive Function (BRIEF-P [26]).

#### 2.2.2. EFs Tests

Pre-post intervention assessments, individually conducted in a quiet room within the school, took about 20 min. The following standardized tests were used.

Simon Says (Italian version of Marshall & Drew’s Simon Says [27]). This test assesses motor inhibition and is composed of two conditions: (A) in the *Activation task,* the child has to autonomously perform only those instructions starting with “Simon says” and ignoring those without; (B) in the *Inhibition task,* the child has to follow the previous rule while the examiner performs all instructions. Accuracy is measured in both conditions.

Day–Night Stroop (FE-PS 2-6 [28]). This test assesses verbal inhibition and is composed of two conditions. In the *Control Accuracy condition,* 16 cards are presented and the child is instructed to say “night” to the card representing a cross and “day” to the card representing a chessboard. In the *Stroop Accuracy condition,* another 16 cards are presented and the child is instructed to say “night” to the card representing the sun and “day” to the card representing the moon. Accuracy and time of response are measured in both conditions.

Keep Truck (FE-PS 2-6 [28]). This test measures updating in verbal working memory. In each trial, six pictures belonging to four possible semantic categories (fruit (banana, pear, strawberry, apple), animals (dog, fish, cat, mouse), transports (train, bicycle, motorcycle, car), clothes (socks, shirt, skirt, shoes) and sky (moon, sun, star, cloud)) are serially presented. The child is instructed to name the pictures and at the end of the trial to recall the last item belonging to a defined category (e.g., “remember the last cloth you will see”). The difficulty increases when the child has to remember simultaneously two stimuli belonging to two different categories. To reduce the memory load, for each picture a small box shows the category(ies) to remember.

Color and Form Game (FE-PS 2-6 [28]). This test measures emerging shifting skills. Stimuli consist of 24 cards showing red or blue rabbits and red or blue boats and two letter boxes depicting, respectively, a red rabbit and a blue boat. The test consists of three conditions. In the *Color condition,* the child is instructed to use the Color criterium and ignoring the Form one (inserting all cards with red pictures in the box depicting the red rabbit and all cards with blue figures in the box depicting the blue boat). In the *Form condition,* the child must use the Form criterion and ignore the Color one (inserting all rabbits into the box depicting a red rabbit and all boats into the box depicting a blue boat). In the *Border condition,* the child is instructed to follow the Color criterium when cards have a black border and the Form criterium when they do not have a border. Accuracy in the Color, Form and Border conditions is measured.

Mr Ant (Italian adaptation by Morra [29]). This test measures updating in visuospatial memory. The examiner presents pictures of peanut-shaped stimuli with a progressively greater number of spots (from 1 to 8). Each picture is shown for a different length of time: 5 s for the first 15 trials, 6 s for the 16–18 trials, 7 s for 19–21 items and 8 s for 22–24 trials. After stimulus presentation, the child has to indicate the spot position on a spotless peanut. The test ends after three consecutive fails. The number of spots correctly localized is measured. The study was approved by the Ethic Direction of the school in September 2018.

### 2.3. Procedure

**Figure 1:**
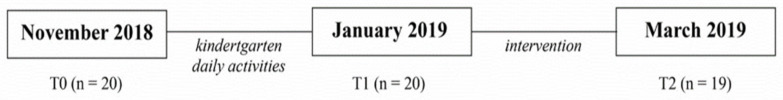
Study Design.

A repeated single sample design was used. Children were assessed in November 2018 (T0), January 2019 (T1) and March 2019 (T2): T1-T0 was the baseline, used as control condition, and T2-T1 was the QQQ_IT_ intervention (see Figure 1).

### 2.4. Intervention

**Figure 2:**
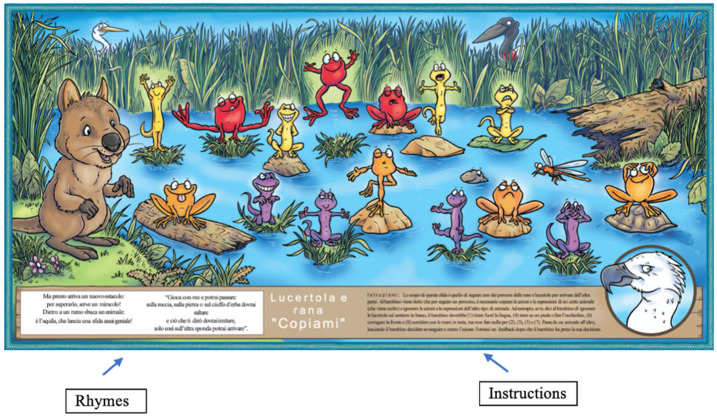
A representation of the QQQ_IT_ activity “Serena Sea Eagle’s. Copy Me!” (modified from Steven Howard & Simon Chadwick [24]).

**Figure 3:**
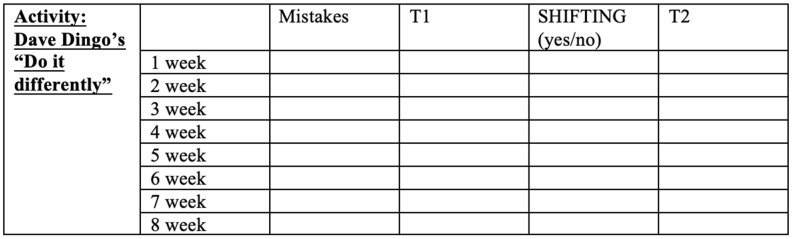
Example of the table format used to monitor the training.

Quincey Quokka’s Quest is an illustrated book that aims to support children’s cognitive development through 8 challenging activities which increase in difficulty. The Italian version of QQQ_IT_ was created by adapting and translating texts, respecting rhymes, while keeping visual and structural features unchanged. The intervention was conducted for 8 weeks, once a week. Activities were executed inside the kindergarten, in a quiet room entirely dedicated to the project. The intervention was carried out in groups of two children, randomly paired each time in order to promote observational and cooperative learning. Each activity was aimed to enhance a specific EF component: updating, inhibition or shifting (see Figure 2). In each session, the child completed from four to five different EF activities, at least one for each EF trained component. The adult did not necessarily need to follow the book’s order as QQQ is a flexible instrument where number, duration or difficulty level of the activities may be changed according to the child’s ability and interest. The weekly intervention lasted about 20 min per group: time taken to carry out a single activity varied from 2 to 4 min, depending on the activity’s nature and on children’s performances. A short time was spent creating an enjoyable atmosphere and reading the rhymes that introduced the activity. In order to propose a challenging exercise, the difficulty level was chosen according to the performances obtained by the child at the previous sessions (see Figure 3). Activities were conducted by two adults or, in the case of one adult, they were video-taped to record the child’s responses without compromising the playful nature of the setting. The adult was carefully trained in the use of QQQ_IT_ and in the EF training principles [30].

A brief description of each QQQ activity follows.

Crocodile! “Cross Back!”: this activity is aimed to train visuospatial memory span, backward and forward. Quincey must cross the river and the adult shows a possible route (e.g., rock, trunk) that the child is invited to copy. As soon as Quincey discovers a dangerous crocodile in front of it on the other riverside, it is forced to go back by doing the inverse route because the only “safe and not wobbly” route is the one where it jumped on earlier. During the following sessions, the adult starts from the reached level.

Cora Copperhead’s “Criss-Cross”: this activity works on shifting. Initially, the child has to find independently the differences among fishes according to three distinctive criteria: color (orange/purple), size (large/small) and skin (dots/stripes). The child is asked to remember two criteria among the three listed above: he/she must use the first criterion to name all the fishes that are in the left circle but as he/she arrives at the first purple fish on the other side, he/she must change the criterion and name all the remaining fishes in this new mode. There can be six levels of increasing difficulty: from level 1 to 3, the stimuli rate increases (from one stimulus every three seconds to one stimulus every second); in the 4th level, the child indicates the fish to each other; in the 5th level, the child conducts the activity on his/her own and in the 6th level the child must change the criterion for each fish.

Serena Sea Eagle’s “Copy Me!”: this activity works on interference control, motor inhibition skills and shifting. In the first condition, the child must copy all the lizards (that are yellow in the first row and purple in the second one), paying attention to their position and facial expression and ignoring frogs (interfering stimuli). In the second condition, the child must copy only frogs (that are red in the first row and orange in the second one) and ignoring lizards (see Figure 2 for a representation of the stimuli). Speed and accuracy are measured. There are three levels of increasing difficulty: (1) the adult touches every stimulus and asks the child whether that stimulus should be copied or not; (2) the child carries out the activity autonomously; (3) the child must copy the stimulus when the adult claps twice and must inhibit copying when the adult claps once. When the activity is conducted in pairs, one child runs the first line, and the other child runs the line below.

Gloria Golden Silk Orb-Weaver’s “List it back”: this activity is aimed to involve visuo-verbal working memory. The adult says that the naughty spider has captured many animals and objects into its web. The child is instructed to free them by remembering their names. The adult names and points to a sequence of animals and objects (starting from 2), covers the book page with a blank paper and asks the child to recall all of them either in the same order (forward span) or backward (backward span). The number of stimuli is progressively increased.

Wesley Wedge-tailed Eagle’s “Say the opposite”: this activity works on verbal inhibition interference control. The picture represents snakes and frogs distributed throughout the pond. To facilitate a visuospatial stimuli organization, different colors indicate different lines. The child should say “snake” when the frog is indicated and “frog” when the snake is indicated. It is possible to alternate animal’s names with sounds, so the child makes “sss” to frog and “cra” to snake. There can be five levels of increasing difficulty: from level 1–3, the stimuli rate increases (from one stimulus every 3 s to one stimulus every second); at level 4, the adult randomly points to different stimuli and at level 5, the child carries out the activity autonomously.

Dave Dingo’s “Do it differently”: this activity is aimed to work on shifting. The child has to use the differences between swamp stones in color (yellow, orange, blue) and shape (square, round, triangle) in order to choose the route to arrive at the river and to change it on the other riverside. For example, he/she may choose the shape before the river and color on the second riverside, or vice versa. The child carries out the activity independently and the adult gives him/her time to reflect on the road’s choice. Speed and accuracy are measured. To increase difficulty, interfering stimuli are introduced: (1) do not touch a stone (e.g., blue square); (2) do not touch two stones (e.g., blue square and orange square).

Cassie Cassowary’s “Chirp Challenge”: It may involve visuo-verbal inhibition. In the picture one or two birds are represented; when the adult points to one bird, the child must make two chirps (“chirp chirp”) while when two birds are indicated, the child must make only one chirp (“chirp”). Colors facilitate the bird’s identification, as birds in pairs have the same color. There are two lines of birds and the child alternates the line when the activity is conducted in pairs. From difficulty level 1 to 3, the stimuli rate increases (from one stimulus every 3 s to one stimulus every second); in the 4th level of difficulty, the adult interferes with the child’s task by saying “chirp” or “chirp chirp” at each stimulus, regardless of the rule.

Quentin Tiger Quoll’s “Animal Spotter”: This activity is aimed to work on updating. It consists of four successive sheets. The purpose of the game is to discover a different animal on each page by avoiding repetitions. As the activity is performed in pairs, recall alternates between children. Accuracy is recorded. Two further activities are possible: at the end of the task, the child is asked to recall the first animal that his/her classmate had chosen or to indicate the spatial position of the animal on a blank sheet.

By a protocol created to record the child’s progress, a QQQ_IT_ Training Monitoring Table, task accuracy and speed, motivation and compliance of the child were recorded each week for each child (see Figure 3). These types of tables, considered as an “in progress inventory”, helped the examiner to plan new challenging activities for the subsequent session and monitor children’s trends.

### 2.5. Statistical Analysis

Training trends and outcome measures were analyzed by descriptive and inferential statistics using the Statistical Package for Social Science 2020, version 25.0 (SPSS, IBM Corporation). Two-tailed paired *t*-tests were used to compare the first and last session scores of the main QQQ_IT_ activities and performances between T1 and T0 (baseline condition) and between T2 and T1 (QQQ _IT_ condition) on the EF tests. Effect size (Cohen’s d) was calculated by G∗Power 3 program [31] between the differences (delta) at the baseline and those at the QQQ _IT_ condition for all EFs tests.

## 3. Results

### 3.1. Socio-Economic and Behavioural EF Profiles

The Socio-Economic and Cultural Status assessment indicated that all children were native Italian speakers, belonged to middle socioeconomic status and had typical development. In detail, all the children (n = 18) had both parents with an occupation, except one child who had one parent unemployed. The level of education of the mothers was at least upper secondary school (n = 19), while in reference to the fathers, all parents had at least a high school diploma except for four parents who had a lower secondary school qualification. Almost all children (n = 18) had both parents born in Italy, except one child whose mother was born in Austria.

As expected on the basis of selection criteria, all children scored within the normal range at BRIEF-P (Table 1).

### 3.2. Qualitative Observations

Teachers, as well as parents, showed interest in the intervention by the QQQ and on the main constructs underlying the project. Although at an informal and not quantified level, both teachers and parents asked questions on the construct of EFs and on the strategies available to early empower them within the daily context.

All children welcomed Quincey’s story with great enthusiasm, actively collaborated and completed the proposed activities, as documented by Training Monitoring Tables. Sometimes children preferred to start with a different activity from the one planned by the trainer, and their request was satisfied. During the session, when the activity seemed to be too difficult for the child, the adult could go back to easier levels to avoid frustration and loss of motivation. As the intervention proceeded, children undertook more proactive roles: some children memorized and repeated the rhyming narratives, others explained task instructions to their schoolmates and helped those who struggled with requests. It was important to give sufficient time to reading and to encourage the child’s expectations and calm. During the activities, the adult was engaged also in reducing distractions and stress factors as much as possible in order to encourage children’s concentration and full cognitive resources’ involvement.

### 3.3. Intervention Trend: Changes in QQQ_IT_ Activities

**Figure 4:**
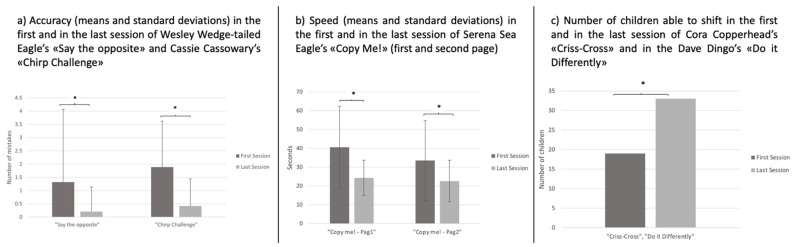
Graphics of the mean, standard deviations, and statistical significance (*t*-test comparison) of the difference between initial and final scores in the main QQQ_IT_ activities. * significant differences.

The quantitative scores recorded by the Training Monitoring Table show a general performances’ improvement in QQQ_IT_ activities from the first to the last sessions. As shown in Figure 4, paired Student *t*-tests showed a significant increase of performances in the last session of the training compared to the first one in the following QQQ_IT_ activities: Wesley Wedge-tailed Eagle’s “Say the Opposite” (t(18) = 2.16, *p* < 0.05), Cassie Cassowary’s “Chirp Challenge” (t(18) = 3.44, *p* < 0.00), Serena Sea Eagle’s “Copy Me!” (Pag1: t(18) = 2.77, *p* < 0.001; Pag2: t(18) = 2.47, *p* < 0.01), and Cora Copperhead’s “Criss-Cross” (chi-quadro(1) = 8.9, *p* < 0.005) and Dave Dingo’s “Do it Differently” (chi-quadro(1) = 11,8, *p* < 0.001) (See Figure 4).

### 3.4. Intervention Effects

Means and standard deviations of raw scores obtained in EF tests at the three time points are reported in Table 2.

**Figure 5:**
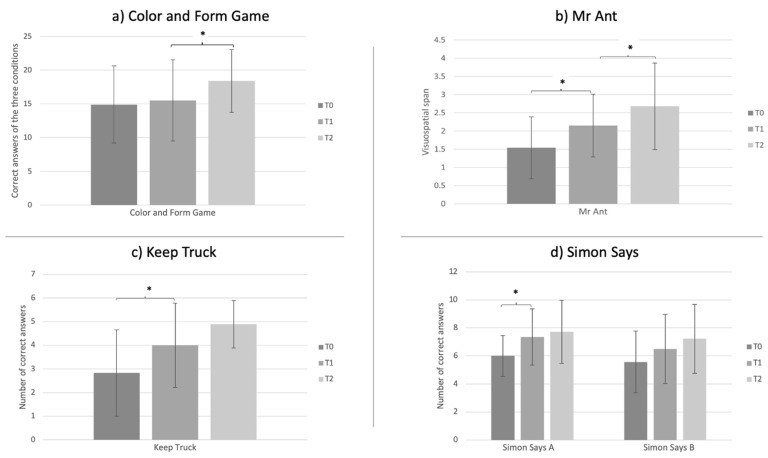
Graphics represents the mean and standard deviations at the three times of observation of the EFs measures showing a significant change between T1-T0 and T2-T1. * significant differences.

At planned *t*-tests, a significant effect of time (T1-T0) was found in Keep Truck Accuracy (t(17) = −2.35, *p* < 0.05), in Simon Says condition A (t(17) = −2.75, *p* < 0.05) and in Mr Ant (t(17) = −3.62 *p* < 0.005) while a significant effect of the training (T2-T1) was found in Color and Form Game Accuracy (t(17) = −2.31; *p* < 0.05) and in Mr Ant (t(17) = −2.87, *p* < 0.05) (Figure 5). These results were confirmed by calculating the Cohen’s effect size. As can be observed from Table 3, large effect sizes in favor of the QQQ_IT_ condition, respect to the baseline, were found for the Form and Color Game test measures except for the Color condition for which the effect size was not calculable because of the absence of variability in the QQQ _IT_ condition. The effect size was not calculable in the Simon Says condition B, and it was small for the Day Night Inhibition condition and for Mr Ant; moderate to large effects were found in favor of the baseline condition in all the other measures.

## 4. Discussion and Conclusions

The main aim of the present study was to verify the feasibility and measure, at a pilot level, the efficacy of the Italian version of Quincey Quokka Quest, a validated training that empowers Executive Functions in preschool children during activities of dialogic reading conducted within the school context. Thanks to the supervision of the authors (Howard and Chadwick), QQQ_IT_ maintains all pictures and EF activities of the original version and the translation to Italian retains the narrative structure of the story and the linguistic form, based on rhymes.

The first result of the study confirms that QQQ_IT_ is a feasible tool to be used in preschools. It has allowed the attraction of parents’ and teachers’ interest in the main constructs of EFs and on the strategies to early empower them within the daily context. By embedding EF exercises in dialogic reading activities, the normal classroom routines were respected without forcing either the child or the adult towards extra-school projects. The feasibility of this ecological approach is in agreement with recent studies highlighting that, especially for preschoolers, working within an ecological context has several advantages such as reduced costs, inclusive approaches, simultaneous involvement of different EF components and high methodological flexibility (e.g., the possibility to choose frequency, duration and daily time of the activities) [9,30]. By combining specific and systematic EF exercises together with a flexible approach, it has been possible to choose the activities according to the child’s preferences and performances, thus avoiding frustration and maintaining high children’s compliance and motivation.

Moreover, performances on QQQ_IT_ activities, at the Training Monitoring Table, showed a systematic trend of improvements that reaches a significant threshold in inhibition speed (Serena Sea Eagle’s “Copy Me!”) and accuracy (Wesley Wedge-tailed Eagle’s “Say the Opposite” and Cassie Cassowary’s “Chirp Challenge”) and in shifting accuracy (Dave Dingo’s “Do it Differently” and “Cora Copperhead’s “Criss-Cross”). These findings confirm that the QQQ training has significant “near” effects, that is, it may improve performances in the executive function tasks trained by this method.

For what concerns the “far effects”, data on the effects of QQQ_IT_ on standardized EF tasks were collected by a protocol of standardized tests proposed before and after the control (baseline) and the experimental (intervention) conditions.

A significant training effect, in comparison to the baseline changes, was found in the Color and Form Game test, which implies the ability to shift among different rules. Thanks to the QQQ_IT_ intervention, children significantly improved their ability in shifting attention and in using flexible classification criteria. This result, which is in agreement with Howard and colleagues’ study [25] may have been even more prompted in our study by encouraging the child to take Quincey’s perspective, to choose the preferred activities and to monitor the other child’s performances. The benefits obtained with QQQ_IT_ intervention on shifting are in line with other previous EF interventions on preschoolers [20,32]. Such an improvement is of paramount importance as shifting supports preschoolers’ approaches to learning and consequently children’s academic preparation [33]. Reaching school-ages with more robust cognitive shifting skills is important as they represent significant predictors of writing and reading comprehension [34,35] and are related to creativity, that is the ability to generate different responses guided by internal stimuli [34] and to the ability to quickly adapt behaviors and responses according to different contexts and environmental demands. According to Magalaes and collaborators’ study, cognitive shifting is a unique predictor of academic achievement above and beyond control variables such as working memory, inhibition, fluid intelligence, attention, planning, especially for students after 4 grades [36]. Moreover, a study conducted by Kertz and collaborators describes as preschoolers with lower cognitive shifting abilities, in comparison to children with high shifting skills balanced for gender, parental education, IQ and severity symptoms at preschool age, showed greater depression and anxiety symptoms in the school years [37].

No training effects, in comparison to the baseline, were found in the other EF measures. Both methodological and theoretical reasons may be found. Alike in Howard and colleagues’ study [25], no significant effects were found on the inhibition tests. This result confirmed that inhibition was not sufficiently empowered by the QQQ training or that more intensive and challenging training is required to foster it and generalize the gains to other inhibition tasks. However, it may also be hypothesized that the Simon Says test might not be sensitive enough to detect changes in inhibitory function: in a recent study conducted by Di Lieto and colleagues [38] on typically developing children attending a first-grade class, a significant effect on inhibition is detected with the Little Frog Test (BIA [39]) but not with Simons Says.

A lack of significant effects was also found in the working memory domain. In verbal working memory (Keep Truck test) no large effects were expected as QQQ activities stress the visuo-spatial elaboration of pictures’ visual characteristics rather than of the verbal characteristics that, indeed, are mainly used to guide the narrative. Moreover, for measures of motor inhibition (Simon Says A), verbal (Keep Truck) and visuospatial (Mr Ant) working memory, significant learning effects were found at the baseline, thus probably thinning the training effects.

Although further studies on larger samples including experimental and control groups are needed, future perspectives may see the application of QQQ_IT_ to children at risk of neurodevelopmental disorders, EF difficulties or low socio-economic level, populations characterized by a week executive function domain [5,40,41]. As pointed out by recent systematic reviews [9,30], EF interventions within the school setting are among the most effective training and thus must be encouraged. Within this perspective, QQQ_IT_ can be a daily ecological way for early prevention in children with EF weakness and a useful tool to enhance school prerequisites. Indeed, it must be acknowledged that differently from standard rehabilitative training or long-term interventions, QQQ is easily usable by teachers as materials and instructions are all included in the book and the activities are not long-lasting. On the other side, these characteristics may explain the small effects found and further longitudinal studies could measure QQQ_IT_ long- term and far effects on other skills such as academic learning and prosocial behavior [42].

## 5. Limits of the Study

The present study was designed as a pilot study, with the aim of evaluating how much training like QQQ can be proposed to preschool children. It has some methodological limitations. Firstly, the absence of a control group and the reduced sample size may have prevented finding large effects or isolate learning from training effects. Moreover, some EF tasks, although taken from standardized batteries, could not be enough sensible to measure training effects and should be re-thought for further studies. Finally, most of the children of the present study belonged to a medium-high socioeconomic level for which, on average, good EFs are expected. Larger effects could in fact be found in children belonging to a low socio-economic environment or with special needs [8,41]. Future studies controlling cognitive and social characteristics of children are needed.

## Figures and Tables

**Table 1:** Means and standard deviations of T scores at BRIEF-P sub-scales (n = 19) (Mean = 50, Standard Deviation = 10).

	M	SD
Inhibition (I)	48.21	9.91
Shift (S)	44.89	6.67
Emotional Regulation (ER)	49.26	8.81
Working Memory (WM)	47.42	9.18
Planning/Organization (PO)	48.21	7.51
Inhibitory Self-Control (ISCI)	48.58	9.69
Flexibility (FI)	45.95	7.18
Emergent Metacognition (EMI)	47.47	8.24
Global Composite Executive Score (GEC)	46.84	8.47

**Table 2:** Means (standard deviation) obtained in the EFs tests before intervention (control condition, T0-T1) and after the intervention (experimental condition, T1-T2).

	T0	T1	T2
Day Night Inhibition Condition	12.61 (4.77)	13.72 (3.04)	14.39 (2.38)
Day Night Accuracy	1.78 (3.34)	1.17 (2.79)	1 (1.97)
Day Night Control Condition Time	57.87 (36.75)	42.86 (18.95)	38.92 (16.12)
Day Night Inhibition Condition Time	53.89 (26.12)	51.96 (23.57)	44.36 (14.73)
Keep Truck Accuracy	2.83 (1.83)	4 (1.78)	4.89 (2.31)
Color and Form Game Color Condition	5.89 (0.47)	5.89 (0.32)	5.83 (0.38)
Color and Form Game Form Condition	4.44 (2.28)	4.56 (2.45)	5.44 (1.54)
Color and Form Game Borders Condition	7 (1.08)	7 (1.78)	8 (2.13)
Color and Form Game Accuracy	14.89 (5.72)	15.5 (6)	18.39 (4.65)
Simon Says Condition A	6 (1.46)	7.36 (2)	7.72 (2.24)
Simon Says Condition B	5.56 (2.2)	6.5 (2.46)	7.22 (2.46)
Mr Ant	1.54 (0.85)	2.15 (0.86)	2.68 (1.19)

**Table 3:** Mean (standard deviation) delta changes during baseline (T1-T0) and experimental (T2-T1) conditions and related effect size in the EFs tests.

	Baseline T1-T0 Mean (SD)	QQQ_IT_ Condition T2-T1 Mean (SD)	Cohen’s d
Day Night Inhibition Condition	1.11 (1.73)	0.67 (0.66)	0.29
Day Night Accuracy	−0.61 (0.54)	−0.17 (0.82)	0.61
Day Night Control Condition Time	−15.01 (17.8)	−3.94 (2.83)	0.79
Day Night Inhibition Condition Time	−1.93 (2.55)	−7.6 (8.84)	0.72
Keep Truck Accuracy	1.17 (0.05)	0.89 (0.53)	0.55
Color and Form Game Color Condition	0.00 (0.15)	−0.06 (0)	NC
Color and Form Game Form Condition	0.12 (0.17)	0.88 (0.91)	0.91
Color and Form Game Borders Condition	0.00 (0.7)	1 (0.35)	1.65
Color and Form Game Accuracy	0.61 (0.28)	2.89 (1.35)	1.85
Simon Says Condition A	1.36 (0.54)	0.36 (0.24)	2.13
Simon Says Condition B	0.94 (0.26)	0.72 (0)	NC
Mr Ant	0.61 (0.01)	0.53 (0.33)	0.25

## Data Availability

The data presented in this study are available on request from the corresponding author.

## References

[1] Zelazo P.D., Müller U. (2007). Executive Function in Typical and Atypical Development. Blackwell Handbook of Childhood Cognitive Development.

[2] Usai M.C., Traverso L., Viterbori P., De Franchi V. (2012). Diamoci una Regolata! Guida Pratica per Promuovere L’autoregolazione a Casa e a Scuola.

[3] Miyake A., Friedman N.P., Emerson M.J., Witzki A.H., Howerter A., Wager T.D. (2000). The Unity and Diversity of Executive Functions and Their Contributions to Complex “Frontal Lobe” Tasks: A Latent Variable Analysis. Cogn. Psychol..

[4] Friedman N.P., Miyake A. (2017). Unity and diversity of executive functions: Individual differences as a window on cognitive structure. Cortex.

[5] Diamond A. (2013). Executive Functions. Annu. Rev. Psychol..

[6] Blair C. (2002). School readiness: Integrating cognition and emotion in a neurobiological conceptualization of children’s functioning at school entry. Am. Psychol..

[7] Alloway T.P., Alloway R.G. (2010). Investigating the predictive roles of working memory and IQ in academic attainment. J. Exp. Child Psychol..

[8] Diamond A., Lee K. (2011). Interventions Shown to Aid Executive Function Development in Children 4 to 12 Years Old. Science.

[9] Scionti N., Cavallero M., Zogmaister C., Marzocchi G.M. (2020). Corrigendum: Is Cognitive Training Effective for Improving Executive Functions in Preschoolers? A Systematic Review and Meta-Analysis. Front. Psychol..

[10] Thorell L.B., Lindqvist S., Nutley S.B., Bohlin G., Klingberg T. (2009). Training and transfer effects of executive functions in preschool children. Dev. Sci..

[11] Bergman Nutley S., Soderqvist S., Bryde S., Thorell L.B., Humphreys K., Klingberg T. (2011). Gains in fluid intelligence after training non-verbal reasoning in 4-year-old children: A controlled, randomized study. Dev. Sci..

[12] Howard S.J., Melhuish E.C. (2017). An Early Years Toolbox (EYT) for assessing early executive function, language, self-regulation, and social development: Validity, reliability, and preliminary norms. J. Psychoeduc. Assess..

[13] Aksayli N.D., Sala G., Gobet F. (2019). The cognitive and academic benefits of Cogmed: A meta-analysis. Educ. Res. Rev..

[14] Howard S.J., Vasseleu E., Batterham M., Neilsen-Hewett C. (2020). Everyday Practices and Activities to Improve Pre-school Self-Regulation: Cluster RCT Evaluation of the PRSIST Program. Front. Psychol..

[15] Marzocchi G.M., Valagussa S. (2011). Le Funzioni Esecutive in Età Evolutiva.

[16] Kusch C.A., Greenberg M.T. (1994). The PATHS Curriculum.

[17] Domitrovich C.E., Greenberg M.T., Cortes R., Kusche C.A. (2005). The Preschool PATHS Curriculum.

[18] Bodrova E., Leong D. (2007). Tools of the Mind: The Vygotskian Approach to Early Childhood Education.

[19] Dias N.M., Seabra A.G. (2016). Intervention for executive functions development in early elementary school children: Effects on learning and behaviour, and follow-up maintenance. Educ. Psychol..

[20] Traverso L., Viterbori P., Usai M.C. (2015). Improving executive function in childhood: Evaluation of a training intervention for 5-year-old children. Front. Psychol..

[21] Traverso L., Viterbori P., Usai M.C. (2019). Effectiveness of an Executive Function Training in Italian Preschool Educational Services and Far Transfer Effects to Pre-academic Skills. Front. Psychol..

[22] Sasser T.R., Bierman K.L., Heinrichs B., Nix R.L. (2017). Preschool Intervention Can Promote Sustained Growth in the Executive-Function Skills of Children Exhibiting Early Deficits. Psychol. Sci..

[23] Cardoso C.D.O., Dias N., Senger J., Colling A.P.C., Seabra A.G., Fonseca R.P. (2018). Neuropsychological stimulation of executive functions in children with typical development: A systematic review. Appl. Neuropsychol. Child.

[24] Howard S.J., Chadwick S. (2015). Quincey Quokka’s Quest: A Picture Book to Support Children’s Cognitive Development.

[25] Howard S.J., Powell T., Vasseleu E., Johnstone S., Melhuish E. (2017). Enhancing preschoolers’ executive functions through embed-ding cognitive activities in shared book reading. Educ. Psychol. Rev..

[26] Gioia G.A., Espy K.A., Isquith P.K. (2014). BRIEF-P Behavior Rating Inventory of Executive Function.

[27] Marshall P.J., Drew A.R. (2014). What makes Simon Says so difficult for young children?. J. Exp. Child Psychol..

[28] Usai M.C., Traverso L., Gandolfi E., Viterbori P. (2017). FE-PS 2-6 Batteria per la Valutazione Delle Funzioni Esecutive in Età Prescolare.

[29] Morra S. (1994). Issues in Working Memory Measurement: Testing for M Capacity. Int. J. Behav. Dev..

[30] Diamond A., Ling D.S., Bunting M., Novick J., Dougherty M., Engle R.W. (2019). Review of the evidence on, and fundamental questions surrounding, efforts to improve executive functions, including working memory. An Integrative Approach to Cognitive and Working Memory Training: Perspectives from Psychology, Neuroscience, and Human Development.

[31] Faul F., Erdfelder E., Lang A.-G., Buchner A. (2007). G*Power 3: A flexible statistical power analysis program for the social, behavioral, and biomedical sciences. Behav. Res. Methods.

[32] Röthlisberger M., Neuenschwander R., Cimeli P., Michel E., Roebers C.M. (2012). Improving executive functions in 5- and 6-year-olds: Evaluation of a small group intervention in prekindergarten and kindergarten children. Infant Child Dev..

[33] Vitiello V.E., Greenfield D.B., Munis P., George J. (2011). Cognitive Flexibility, Approaches to Learning, and Academic School Readiness in Head Start Preschool Children. Early Educ. Dev..

[34] Filippetti A.V., Krumm G. (2020). A hierarchical model of cognitive flexibility in children: Extending the relationship between flexi-bility, creativity and academic achievement. Child Neuropsychol..

[35] Hung C.O.Y., Loh E.K.Y. (2020). Examining the contribution of cognitive flexibility to metalinguistic skills and reading compre-hension. Educ. Psychol..

[36] Magalhães S., Carneiro L., Limpo T., Filipe M. (2020). Executive functions predict literacy and mathematics achievements: The unique contribution of cognitive flexibility in grades 2, 4, and 6. Child Neuropsychol..

[37] Kertz S.J., Belden A.C., E Tillman R., Luby J.L. (2016). Cognitive Control Deficits in Shifting and Inhibition in Preschool Age Children are Associated with Increased Depression and Anxiety Over 7.5 Years of Development. J. Abnorm. Child Psychol..

[38] Di Lieto M.C., Pecini C., Castro E., Inguaggiato E., Cecchi F., Dario P., Cioni G., Sgandurra G. (2020). Empowering Executive Functions in 5- and 6-Year-Old Typically Developing Children Through Educational Robotics: An RCT Study. Front. Psychol..

[39] Marzocchi G.M., Re A.M., Cornoldi C. (2010). BIA. Batteria Italiana per l’ADHID per la Valutazione dei Bambini con Deficit di Attenzione-Iperattività. Con DVD e CD-ROM.

[40] Paananen M., Aro T., Närhi V., Aro M. (2017). Group-based intervention on attention and executive functions in the school context. Educ. Psychol..

[41] John A.M.S., Kibbe M., Tarullo A.R. (2019). A systematic assessment of socioeconomic status and executive functioning in early childhood. J. Exp. Child. Psychol..

[42] Bryce D., Whitebread D., Szűcs D. (2015). The relationships among executive functions, metacognitive skills and educational achievement in 5 and 7 year-old children. Metacognition Learn..

